# Determinants of Sports Injury in Young Female Swedish Competitive Figure Skaters

**DOI:** 10.3389/fspor.2021.686019

**Published:** 2021-06-18

**Authors:** Moa Jederström, Sara Agnafors, Christina Ekegren, Kristina Fagher, Håkan Gauffin, Laura Korhonen, Jennifer Park, Armin Spreco, Toomas Timpka

**Affiliations:** ^1^Department of Health, Medicine and Caring Sciences, Athletics Research Center, Linköping University, Linköping, Sweden; ^2^Division of Children's and Women's Health, Department of Biomedical and Clinical Sciences, Linköping University, Linköping, Sweden; ^3^Rehabilitation, Ageing and Independent Living (RAIL) Research Centre, School of Primary Allied Health Care, Monash University, Melbourne, VIC, Australia; ^4^Rehabilitation Medicine Research Group, Department of Health Sciences, Lund University, Lund, Sweden; ^5^Departments of Orthopedics and Biomedical and Clinical Sciences, Linköping University, Linköping, Sweden; ^6^Departments of Child and Adolescent Psychiatry and Biomedical and Clinical Sciences, Center for Social and Affective Neuroscience, Linköping University, Linköping, Sweden; ^7^Department of Surgery, Institute of Clinical Sciences, University of Gothenburg, Sahlgrenska Academy, Gothenburg, Sweden; ^8^Department of Health, Medicine and Caring Sciences, Division of Society and Health, Linköping University, Linköping, Sweden; ^9^Center for Health Services Development, Region Östergötland, Linköping, Sweden

**Keywords:** figure skating, sports injury, overuse injury, athletic injury, epidemiology, adolescent, child, pediatric

## Abstract

**Introduction:** Although figure skating attracts several hundred thousand participants worldwide, there is little knowledge about physical health and sports injuries among young skaters. The present study aimed to describe the health status of a geographically defined Swedish population of licensed competitive figure skaters and to examine injury determinants.

**Methods:** All licensed competitive skaters in the southeastern region of Sweden were in April 2019 invited to participate in a cross-sectional study using an online questionnaire. Multiple binary logistic regression was used for the examination of injury determinants. The primary outcome measure was the 1-year prevalence of a severe sports injury episode (time loss >21 days). The secondary outcome measure was the point prevalence of an ongoing injury. The determinants analyzed were age, skating level, relative energy deficiency indicators, and training habits.

**Results:** In total, 142 (36%) skaters participated, 137 (96%) girls [mean (SD) age: 12.9 (SD 3.0) years]. Participating boys (*n* = 5) were excluded from further analysis. The 1-year prevalence of a severe sports injury episode was 31%. The most common injury locations for these injuries were the knee (25%), ankle (20%), and hip/groin (15%). In the multiple model, having sustained a severe injury episode was associated with older age (OR 1.2, 95% CI 1.1–1.4; *p* = 0.002) and an increased number of skipped meals per week (OR 1.1, 95% CI 1.0–1.3; *p* = 0.014). The point prevalence of an ongoing injury episode was 19%. The most common locations were the knee (24%), ankle (24%), and foot (24%). Having an ongoing injury episode was associated with older age (OR 1.4, 95% CI 1.2–1.7; *p* < 0.001) and an increased number of skipped meals per week (OR 1.1, 95% CI 1.0–1.3; *p* = 0.049).

**Conclusion:** One-third of young female Swedish competitive figure skaters had sustained a severe injury episode during the past year, and a fifth reported an ongoing episode. Older age and an increased number of skipped meals per week were associated with a sports injury episode. Long-term monotonous physical loads with increasing intensity and insufficient energy intake appear to predispose for injury in young female figure skaters. Further examination of injury determinants among competitive figure skaters is highly warranted.

## Introduction

Figure skating was one of the first sports to be included in the Olympic Winter Games (Olympic Games, 2020). Despite the positive effects of physical activity and figure skating, adolescent, and adult aesthetic athletes (such as figure skaters, ballet dancers, and rhythmic gymnasts) have been reported to be at increased risk of developing health problems, e.g., injuries, eating disorders, anxiety, and depression (Smolak et al., 2000; Rosendahl et al., 2009; Krentz and Warschburger, 2013; Mayolas-Pi et al., 2021). In Sweden (population 10.2 million), figure skating attracted more than 30,000 participants in the 2018–2019 season (Swedish Figure Skating Association, 2018). Around 80% of these skaters were ≤ 25 years old; 17% were competitive skaters (Swedish Figure Skating Association, 2018), of which 74 (1%) were competitive elite figure skaters on junior and senior level (Swedish Figure Skating Association, 2020). One leading health problem that causes concern among figure skaters is injury. Most published studies on figure skating injuries have been based on national or international elite skaters (Ziegler et al., 1998; Ziegler P. et al., 2002; Ziegler P. J. et al., 2002; Dubravcic-Simunjak et al., 2003; Fortin and Roberts, 2003). However, figure skating is often referred to as an “early entrance sport,” as it is considered necessary to master technique and difficult elements before puberty (Smith, 2000). This requires extensive training hours at an early age, often by early specialization (Monsma, 2008). Nonetheless, most young skaters will never reach the elite level, and thus previous research is not generalizable to most participants (Jaworski and Ballantine-Talmadge, 2008). Knowledge about sports-related health threats in adults is not directly applicable to young children and adolescents, and the research on the incidence and prevalence of overuse injuries in children and adolescents is scarce (DiFiori et al., 2014). Moreover, studies on child athletes' injuries are based on observations from team sports such as football and field hockey, and there is limited research available on the risks associated with individual sports (Steffen and Engebretsen, 2010; Dahlström et al., 2012).

Injury risk in child athletes may be affected by numerous intrinsic and extrinsic factors, ranging from competitive level, training load, and equipment to eating and sleeping habits (West et al., 2021). Sleep deprivation is, for example, associated with reduced reaction time, performance, motivation, and mood changes (Mah et al., 2011; Halson, 2014), and chronic lack of sleep is associated with sports injuries in adolescents (Milewski et al., 2014). Socioeconomic status is also associated with sports injuries among children and adolescents through mechanisms such as being brought up to keep playing and practicing throughout pain and poor equipment (Dahlström et al., 2012). From previous studies on elite figure skaters, overuse injuries appear to be more common than traumatic injuries, and there seems to be a connection between increased training hours in figure skating and sports injuries (Dubravcic-Simunjak et al., 2003). Overuse injuries have risk factors that are both intrinsic (for example, growth spurt and menstrual dysfunction) and extrinsic (for example, training load and training schedule; DiFiori et al., 2014). Also, in figure skating, overuse-related conditions associated with a bad boot fit appears to be common (Campanelli et al., 2015).

A particular cluster of health problems causing concern in figure skating is body and weight dissatisfaction (not being content with the way your body looks and not being content with your weight, respectively; Monsma et al., 2006; Monsma, 2008), eating disorders (a diagnosis fulfilling the DSM-V or ICD-10 criteria), and the Relative Energy Deficiency in Sport (RED-S) syndrome (Rosendahl et al., 2009; Van Durme et al., 2012; Krentz and Warschburger, 2013; Voelker et al., 2014). Low energy availability (LEA) is the aetiological factor of the RED-S syndrome and is also recognized as a risk factor for overuse injury (Mountjoy et al., 2018). Female figure skaters may have more eating pathology than female adolescents from a general population (Van Durme et al., 2012), and elite figure skaters have been found to consume an inadequate amount of energy throughout the day (Ziegler P. J. et al., 2002). Also, aesthetic sports have previously been linked to an increased risk of disordered eating (subclinical conditions that are in between healthy and pathological eating patterns and may lead to adverse effects on health; Sundgot-Borgen and Torstveit, 2010), body dissatisfaction, anxiety and depression symptoms and poor sleeping habits (such as subjective low sleep quality, low sleep duration, and sleep disturbances; Smolak et al., 2000; Rosendahl et al., 2009; Krentz and Warschburger, 2013; Mayolas-Pi et al., 2021). Regarding the problem cluster including body and weight dissatisfaction, eating disorders, and the RED-S syndrome, there is very little knowledge available about the prevalence of these factors in child- and adolescent athletes and whether there are any associations with injuries.

Given the lack of research on injuries and athlete health in community-level figure skaters, this study aimed to describe the general health status and lifestyle issues of a geographically defined Swedish population of licensed competitive figure skaters and thereafter to examine injury determinants.

## Methods

### Study Design

This study employed a cross-sectional design with data collection using an online questionnaire. The primary and the secondary outcome measures were defined in terms of injury or pain that had occurred in connection with training or competition in figure skating, which prevented the skater from fully participating in training or competition in figure skating. The primary outcome measure was self-report of having sustained a severe injury episode in the last 12 months. The secondary outcome measure was self-report of an ongoing injury episode.

### Population

The primary study population consisted of the total population of licensed competitive figure skaters from the Swedish Southeastern Regional Figure Skating Federation (population of 400 competitive skaters in the 2018–2019 season), which is part of the Swedish Figure Skating Association (population of 5,000 competitive skaters in the 2018–2019 season).

### Study Sample

The 10 figure skating clubs with licensed competitive skaters in Sweden's southeastern region agreed to invite their competitive skaters (*N* = 400) to participate in the study. Information meetings about the study were arranged at each club in the region by MJ. The skaters and parents of skaters younger than 15 years also received written study information. The figure skating clubs were asked to collect email-addresses to help administer the survey. After that, an email with information about the study and an invitation to participate was sent via figure skating clubs to all guardians of skaters younger than 15 years old and all skaters 15 years or older.

### Data Collection

Data were collected between April and July 2019 using online surveys sent through the online-questionnaire-system Briteback?. Informed consent was collected from parents (if the skater was below 15 years of age) and all skaters in the first part of the questionnaire (Appendix I in Supplementary Material).

The skaters were expected to answer the questions by themselves. Guardians of children younger than 15 years were strongly advised not to supervise their children while answering but to help their children interpret questions if they asked for help. Participants were asked to complete a questionnaire designed for their age group (≥/ <12 years of age). Each figure skating club was asked to send four reminders during the response time.

The participating figure skating clubs were asked to contribute aggregated data about their competitive skaters at the survey time. This included anonymous information regarding sex, date of birth (year and month) and competitive level.

### Online Questionnaire

The online questionnaire (Appendix I in Supplementary Material) was designed based upon questionnaires previously used with young athletics athletes, football (soccer) players and parasport athletes (Jacobsson et al., 2010, 2012; Dahlström et al., 2012; Fagher et al., 2020). Also, questions from the 2014 study Health Behaviors in School-aged Children (HBSC) by the Public Health Agency of Sweden were used, including questions about the socioeconomic environment, physical activity, eating behaviors, sleeping habits, body image, weight reduction behavior, long-term illness or other health problems, drug use, self-rated health, and social support (Public Health Agency of Sweden, 2014). The entire survey was estimated to take 20 min to complete.

The questionnaire was tested and evaluated by the first author (MJ), participants of the research group, physicians from the Swedish Figure Skating Association (SA & JP), former competitive figure skaters and children who were not yet eligible for competition (in order to test how younger skaters perceived the questions). Feedback was also requested from parents of children below 15 years of age after testing the questionnaire.

The questionnaire contained mostly closed items (tick-the box format) with a limited number of free-text questions. Only questions about key outcome variables were marked as compulsory. Skater characteristics collected included age, height, weight, eating, and sleeping habits, growth in the past year, social support, bullying and socioeconomic factors (parents' country of origin, housing, parents' level of education, and the family's financial situation). Questions on sport-specific characteristics included items on training and competition habits, competitive level, skating level, jumping direction, use of protection/shock absorption, use of aids, skate brand, and if the skater had customized their skates in any way. Questions on health status included physical health (menarche, irregular menstruation, chronic illness, and use of medication) and mental health (well-being, anxiety, self-rated health, body image, weight concern, dieting, and if the skater had been asked to adjust his or her weight).

Regarding sports injuries, questions were asked about a severe sports injury episode sustained the past 12 months and an ongoing sports injury episode. The definition of a severe sports injury episode was any injury or pain that had occurred in connection with training or competition in figure skating, which resulted in >21 days of lost or altered participation in figure skating (Darrow et al., 2009). An ongoing sports injury episode was defined as an injury or pain that had occurred in connection with training or competition in figure skating, which prevented the skater from fully participating in training or competition at the survey time. If the skater had sustained an injury episode, he/she was asked about the injury mechanism (overuse/trauma). An overuse injury was defined as an injury or pain that arose through repeated overload. The definition of trauma was if the injury or pain occurred due to external force on one occasion, for example, if the skater got stuck with the foot in the ice. Thereafter, the skater was asked to report the affected body part(s). Body regions for injuries were defined using the OSIICS recommended categories of body regions and areas for injuries (Bahr et al., 2020).

### Data Analysis

The data on skater characteristics were analyzed descriptively using percentages for categorical variables and mean values and standard deviations (SD) for continuous variables. Body Mass Index (BMI: body mass in kg divided by the square of the body height) was presented as International Obesity Task Force-BMI (IOTF-BMI) for respondents below 18 years of age (Cole and Lobstein, 2012). Indicators of RED-S were represented in terms of the skater being underweight according to IOTF-BMI OR reporting irregular menstruation (yes/no). A categorical variable was constructed for sleeping hours by multiplying sleeping time on weekdays by five and sleeping time on weekends by two. These data were then categorized as “too little sleep,” “enough sleep,” and “too much sleep,” based on the Swedish recommendations on sleeping hours per night for different age groups (children age 6–12: 10–11 h per night; adolescents age 13–18: 8–9 h per night; adults: 7–9 h per night; Swedish Public Health Recommendations, 2020). The variable skating level was recoded from four categories [attainment of up to single axel/single axel & 1 double jump/all double jumps (including double axel/double axel & 1 triple jump); Campanelli et al., 2015] to two categories (attainment of up to double toeloop/double loop and higher; Appendix II in Supplementary Material).

**Figure 1:**
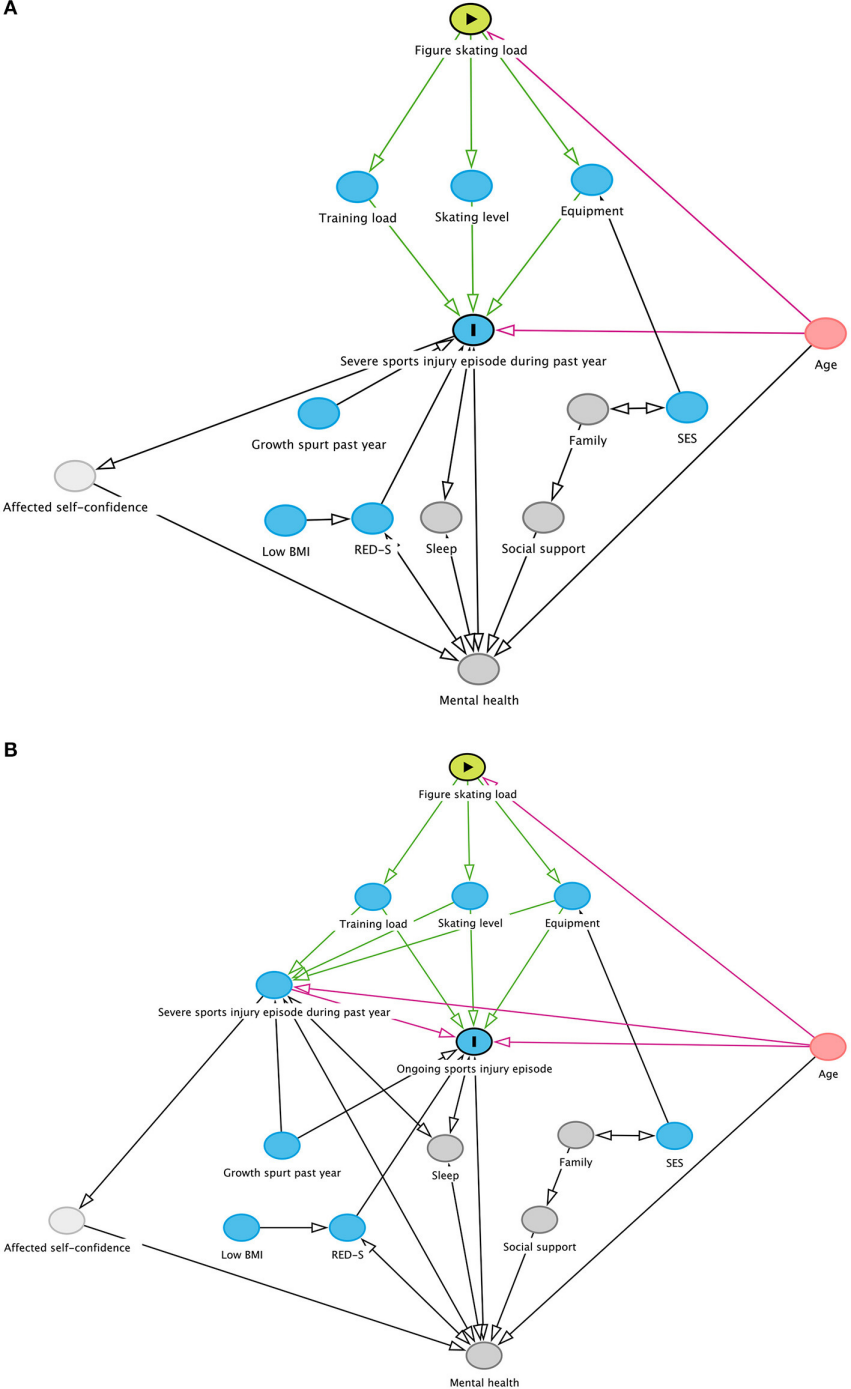
Theoretical representation of health issues in figure skating. BMI, body mass index; SES, socioeconomic status; RED-S, relative energy deficiency in sport; Blue node, outcome and ancestors of the outcome; Green node, exposure; Pink node, ancestor of exposure and outcome; Dark gray node, other variables; Light gray node, unobserved (latent); Green arrow, casual path; Pink arrow, biasing path. **(A)** Outcome: Severe sports injury episode during the past year. **(B)** Outcome: Ongoing sports injury episode. Graphs created using DAGitty.

To inform the choice of variables to be included in the analyses of injury determinants, a theoretical representation of injury mechanisms in figure skating was developed based on the current literature (Figure 1; Dubravcic-Simunjak et al., 2003; Mah et al., 2011; Dahlström et al., 2012; DiFiori et al., 2014; Halson, 2014; Milewski et al., 2014; Campanelli et al., 2015; West et al., 2021). The primary outcome variable represented self-report of a severe sports injury episode in the last 12 months (yes/no) (Figure 1A), and the secondary outcome variable report of an ongoing sports injury episode (yes/no) (Figure 1B). The explanatory variables included in the model of injury determinants were age (continuous), the number of skipped meals per week (continuous), RED-S-indicators (yes/no), skating level (up to double toeloop/double loop and higher; not included for ongoing injury episode) and mean weekly training hours (up to 6/7–9/10 h or more). Separate analyzes will be performed regarding mental health.

First, simple binary logistic regression analyses were performed to determine factors associated with the primary and secondary outcomes. Next, multivariable models were fitted using backward elimination of non-significant variables (i.e., variables with *p* ≥ 0.05 were stepwise eliminated). Nagelkerke *R*^2^ was obtained for the multivariable model to estimate its accountability level. All statistical tests were two-sided, and associations with *p* < 0.05 were considered to be statistically significant. The Statistical Package for the Social Sciences (SPSS) for Windows version 26.0 was used for all analyses.

## Results

**Figure 2:**
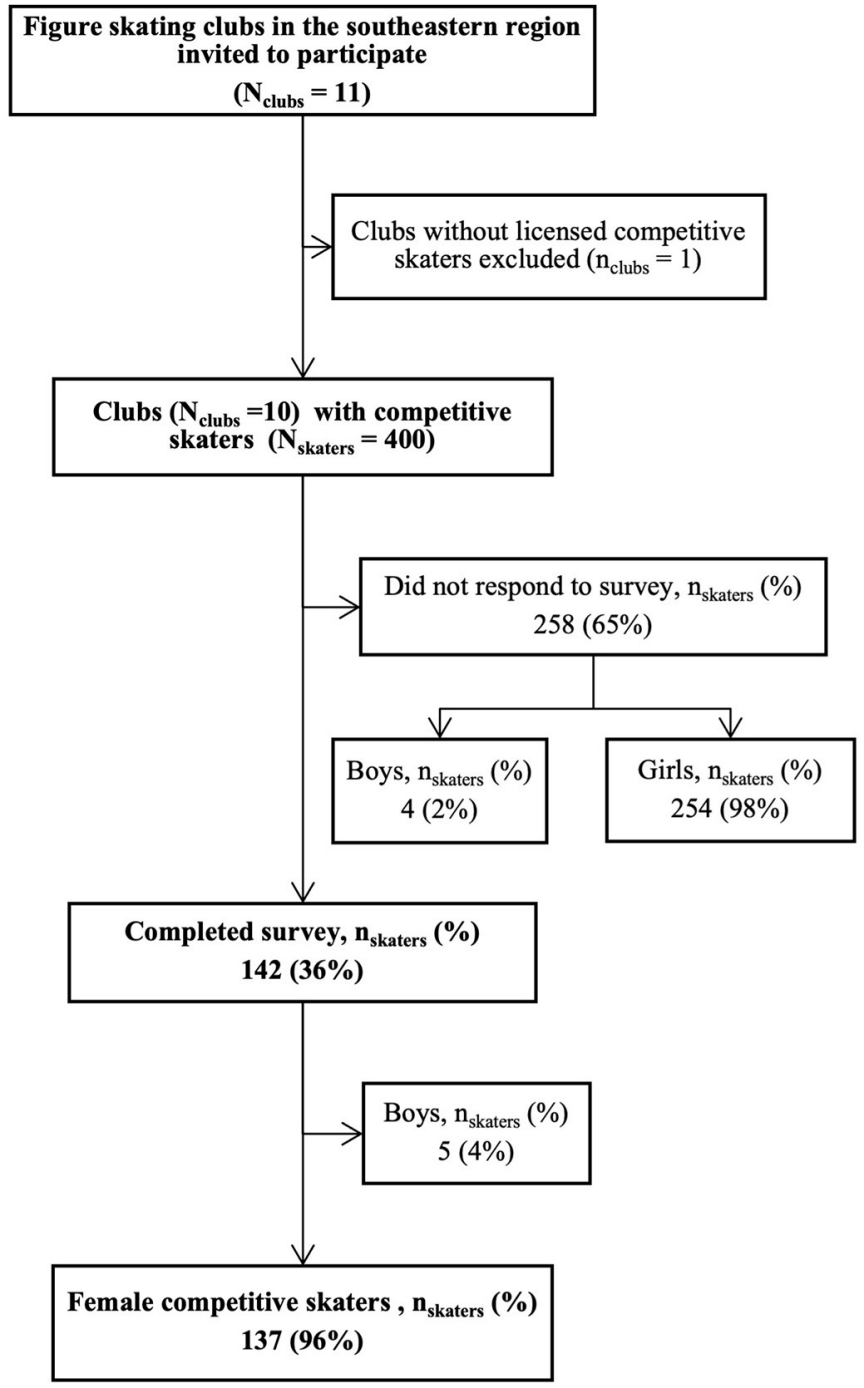
Study recruitment flow chart.

A total of 142 (36%) skaters completed the survey, of whom 137 (96%) were girls, and five (4%) were boys (Figure 2). Skaters who participated in the study differed from the population invited according to competitive level (*p* < 0.001), but not according to sex (*p* = 0.203) or birthyear (*p* = 0.173). Skaters from club-competitions-level were overrepresented among the respondents, while skaters from star-competitions-level were underrepresented (Appendix III in Supplementary Material). Boys (*n* = 5) were excluded from analysis due to the potential for identification.

### Study Population

The mean age of the 137 female skaters included in the analysis was 12.9 (SD 3.0) years. Some (17%) were elite skaters or skating at a national level (A-level), 45% were at club-competition level, and 39% were at the star-competition level. Regarding skating level, 37% had landed jumps up to double toeloop, and 63% had landed double loop or more advanced jumps. Many (56%) of the female skaters were exercising 7 h per week or more (outside school hours). Most (72%) were practicing figure skating between 4 and 9 h each week, and the majority (73%) were resting 1 or 2 days each week (Table 1).

**Table 1:** Characteristics of study population (*n* = 137).

	***n* (%)**
**Age**	
Mean (SD)	12.9 (3.0)
<12 years	47 (34%)
12–15 years	68 (50%)
>15 years	22 (16%)
**Competitive level**	
Elite skater/A-competitions	23 (17%)
Club competitions	61 (45%)
Star competitions	53 (39%)
**Growth (in height) in the past year**	
0–5 cm	91 (66%)
6–10 cm	41 (30%
>10 cm	5 (4%)
**Discipline**	
Singles skating	137 (100%)
**Jumping direction**	
Right (landing on left left)	21 (15%)
Left (landing on right leg)	116 (85%)
**Skating level**	
Landed up to double toe loop	51 (37%)
Landed double loop and higher	86 (63%)
**Total exercise per week (hours)**	
≤ 1 h	5 (4%)
2–3 h	11 (8%)
4–6 h	44 (32%)
7–9 h	35 (26%)
10–12 h	28 (20%)
≥13 h	14 (10%)
**Figure skating per week (hours)**	
2–3 h	12 (9%)
4–6 h	67 (49%)
7–9 h	32 (23%)
10–12 h	19 (14%)
≥13 h	7 (5%)
**Resting days per week in the last 12 months (means)**	
0 days	6 (4%)
1–2 days	100 (73%)
3–6 days	31 (23%)

The majority (82%) were using Edea skates. Most (63%) were 6 years or younger when they started skating. Some (35%) were participating in other sports, and about a third (36%) had participated in six or more competitions in the past year (Appendix IV in Supplementary Material).

### Health Status and Lifestyle Issues

About a tenth of participants (12%) were, according to self-reported data, underweight, and 4% were overweight. About half (48%) had reached menarche, of which 42% had irregular menstruation. More than one-third of the female skaters (38%) were, according to Swedish public health recommendations, getting too little sleep each week, and a few (17%) were getting too much sleep each week. Most (82%) were eating breakfast every weekday, and 90% were eating breakfast every weekend (Table 2).

**Table 2:** Health and lifestyle characteristics of participants (*n* = 137).

	***n* (%)**
IOTF- BMI^*^	
Underweight (IOTF-BMI ≤ 18.5)	16 (12%)
Normal	111 (81%)
Overweight (IOTF-BMI ≥ 25)	5 (4%)
**Menarche**	
No	71 (52%)
Yes	66 (48%)
**Menarcheal age (*****n*** **=** 66)^**^	
≤ 11 years	12 (18%)
12 years	15 (23%)
13 years	23 (35%)
≥14 years	13 (20%)
**Irregular menstruation**	
Yes	28 (20%)
No/not menarche	109 (80%)
**Long-term illness/health problem specified as**	
**(multiple options possible**, ***n*** **=** **52)**	
No	104 (76%)
Yes	33 (24%)
Allergy	26 (50%)
Eczema	8 (15%)
Asthma	12 (23%)
Other	6 (12%)
Current health issue^*^	
Yes	7 (5%)
No	125 (91%)
**Drug use last month**	
No	54 (39%)
Yes	83 (60%)
**Drug (multiple options possible)**	
Painkillers (for example, paracetamol)	54 (39%)
Anti-inflammatory drugs (for example, NSAID)	23 (17%)
Medicines for allergy	36 (26%)
Contraceptive pills	9 (7%)
Other	8 (6%)
**Amount of sleep per week**	
Too little sleep	52 (38%)
Enough sleep	61 (45%)
Too much sleep	24 (17%)
**NUMBER OF SKIPPED MEALS ON WEEKDAYS**	
**Breakfast**	
None	113 (82%)
≥1 weekday(s)	24 (18%)
**Lunch**	
None	118 (86%)
≥1 weekday(s)	19 (14%)
**Dinner**	
None	121 (88%)
≥1 weekday(s)	16 (12%)
**NUMBER OF SKIPPED MEALS ON WEEKENDS**	
**Breakfast**	
None	123 (90%)
≥1 day(s)	14 (10%)
**Lunch**	
None	129 (94%)
≥1 day(s)	8 (6%)
**Dinner**	
None	133 (97%)
≥1 day(s)	4 (3%)

*IOTF-BMI, international obesity task force body mass index*.

* *Five missing responses*.

** *Four missing responses*.

### Injury Prevalence

The 1-year prevalence of a severe sports injury episode was 31% (*n* = 42 participants). Of the female skaters reporting a severe injury episode, 64% had sustained an overuse injury and 36% a traumatic injury. The most common severe injury locations according to the OSIICS categories were the knee (25%), ankle (20%), and hip/groin (15%).

The point prevalence of an ongoing sports injury episode was 19% (*n* = 26 participants). Of the female skaters reporting an ongoing episode, 81% had a current overuse injury, and 19% a traumatic injury. The most common locations of a current injury according to the OSIICS categories were the knee (24%), ankle (24%), and foot (24%).

### Injury Determinants

The simple binary logistic regression analyses showed that having a severe injury episode was associated with older age, increased number of skipped meals per week, and the female skater being underweight or having irregular menstruation (having a RED-S-indicator). In the multiple model, only older age and an increased number of skipped meals per week remained as determinants (Nagelkerke *R*^2^ = 0.17; Table 3).

**Table 3:** Injury determinants for the primary outcome (severe injury episode in the past 12 months, *n* = 42) and secondary outcome (ongoing injury episode, *n* = 26).

**Explanatory variables**	**Simple models**	**Multiple model**
	**Crude odds ratio (95% confidence interval)**	**Odds ratio (95% confidence interval)**
**Severe injury episode in the last 12 months**		
**(Nagelkerke** ***R***^**2**^ **=** **0.17)**		
Age	1.2 (1.1–1.4); *p* = 0.002	1.2 (1.1–1.4); *p* = 0.002
Skipped meals per week	1.1 (1.0–1.3); *p* = 0.010	1.1 (1.0–1.3); *p* = 0.014
RED-S-indicators present	2.5 (1.1–5.4); *p* = 0.021	–
Skating level	1.5 (0.7–3.2); *p* = 0.314	–
**Mean weekly training hours**		
≤ 6 h (reference category)	*p* = 0.578	–
7–12 h	0.9 (0.4–2.0); *p* = 0.862	–
≥13 h	1.8 (0.5–5.8); *p* = 0.358	–
**Ongoing injury episode**		
**(Nagelkerke** ***R***^**2**^ **=** **0.25)**		
Age	1.4 (1.2–1.7); *p* <0.001	1.4 (1.2–1.7); *p* <0.001
Skipped meals per week	1.1 (1.0–1.2); *p* = 0.040	1.1 (1.0–1.3); *p* = 0.049
RED-S-indicators present	1.1 (0.4–2.8); *p* = 0.845	–
**Mean weekly training hours**		
≤ 6 h (reference category)	*p* = 0.599	–
7–12 h	1.2 (0.5–3.0); *p* = 0.731	–
≥13 h	2.0 (0.5–7.7); *p* = 0.312	–

Regarding ongoing injuries, the simple binary logistic regression analyses showed that an ongoing episode was associated with older age and an increased number of skipped meals per week. Both variables remained in the multiple model (Nagelkerke *R*^2^ = 0.25; Table 3).

## Discussion

This study describes the health status of a geographically defined Swedish population of female licensed competitive figure skaters and thereafter examines injury determinants. We report that one-third of the participating young female skaters had sustained a severe sports injury episode during the past year, and a fifth suffered an ongoing sports injury episode. For both severe and current injuries, overuse was the main cause, and the lower limbs were the most common locations. Older age and an increased number of skipped meals per week were associated with both a severe sports injury episode in the last 12 months and an ongoing injury episode. Indicators of RED-S (in terms of the skater being underweight or reporting irregular menstruation) was associated with a severe sports injury episode in the simple models, but not in the respective multiple models.

The injury prevalence in figure skating has been reported to have increased over the last 50 years due to increased technical demand and increased training hours (Jaworski and Ballantine-Talmadge, 2008). In 2003, 43% of female elite junior figure skaters described having sustained overuse syndromes sometime during their skating career (Dubravcic-Simunjak et al., 2003). A recent retrospective study on figure-skating related injuries among 294 American figure skaters between 9 and 19 years of age found that 69% of the injuries were due to overuse and 31% were traumatic (Kowalczyk et al., 2019), similar to the results in this study. An overuse injury may negatively affect future sports participation and result in long-term health consequences (DiFiori et al., 2014) and is recognized to affect those exposed to repetitive loading combined with inadequate rest. At the same time, young athletes may have difficulty recognizing fatigue or poor performance as a sign of an injury (Brenner, 2007). Campanelli et al. (2015) have reported that both young figure skaters and their coaches often overlook pain in the lower extremities. In Italian rhythmic gymnastics, there are findings of a culture of risk; in which athletes have been found to accept pain as a part of the sport, continues to practice and compete despite experiencing pain, and are being punished by the coaches when not adhering to the “mental toughness” that is promoted in the club (Cavallerio et al., 2016). These observations suggest that it is of utmost importance that adults in the skater's environment know about the susceptibility to injuries among young athletes, the necessity of resting when a skater experiences pain, and emphasize a gradual rehabilitation toward returning to full-time practice.

As in previous studies about figure skating (Han et al., 2018; Kowalczyk et al., 2019), the lower limbs seem to be more affected by an injury. This reflects figure skating's biomechanics, where the elements performed require work mainly in the foot/ankle, knee, and hip joints. Also, skates restrict plantar flexion when jumping, which have been proposed to redistribute the energy produced by the knee extensors to the hip and the ankle joints, leading to an increased risk of injury (Haguenauer et al., 2006). The skating boot might also cause tendon- and ligament injuries in the foot/ankle (Kowalczyk et al., 2019). Moreover, asymmetrical loading in the joints of the same leg during repeated high impact landings might further increase the injury risk.

A tenth of the respondents was classified as being underweight (based on self-reported data), and almost half of the female skaters that had reached menarche reported irregular menstruation. Irregular menstruation is quite common in the years following menarche and is associated with polycystic ovary syndrome and endometriosis. However, irregular menstruation and underweight may also appear after a period with energy deficiency (LEA) and thus be a part of the RED-S syndrome. Relative energy deficiency is a severe condition associated with multiple adverse health consequences such as increased injury risk, decreased training response, impaired judgement, osteoporosis, and fertility disturbances (Milewski et al., 2014). Energy deficits should be addressed via modification of exercise and nutrition (Mountjoy et al., 2018). It is critical with early detection of athletes with or at risk for energy deficiency and the involvement of adequately trained experts (for example, sports dieticians).

Regarding injury determinants, older age and an increased number of skipped meals per week were in the multiple models found to be associated with both a severe injury episode in the last 12 months and an ongoing episode. In the age interval covered by this study (6–23 years), older age is associated with higher physical capacity. The elite female figure skaters are today landing quadruple jumps. Landing these higher revolution jumps creates large forces which are transmitted upwards along the kinetic chain. It is currently unknown how these demands affect the prevalence and rate of injury. The association between age and injury may also be explained by that older athletes were more likely to have persisting injury problems, i.e., inadequately managed injuries that become chronic in nature (Jacobsson and Timpka, 2015). Moreover, the association between skipped meals and injury indicates that some female skaters had an energy intake that was insufficient to support the functions required by the body to maintain optimal health and performance [Low Energy Availability (LEA); Mountjoy et al., 2018]. Not consuming an adequate amount of energy puts the body at risk for reduced bone health, reproductive health, hormonal health, and mental health (Ziegler P. J. et al., 2002; Bergeron et al., 2015; Mountjoy et al., 2018). Persistent LEA may also impair sports performance, for example, through impaired recovery leading to a premature reduction in physical, psychological, and mental capacity and impairment of optimal muscle mass and function (Mountjoy et al., 2018). In elite rhythmic gymnasts, athlete performance (competition ranking) is negatively correlated with energy availability (Silva and Paiva, 2016).

Indicators of RED-S (in terms of the skater being underweight or reporting irregular menstruation) was associated with severe injury in the simple models, but not in the multiple model. It may be that the RED-S indicators were displaced from the multiple models by the skipped meals variable, i.e., by a more distal cause in the etiological chain leading to injury. It may also be that the observed underweight and irregular menstruation can in part be explained by variations in growth and development among the female skaters. The associations between energy intake, being underweight, irregular menstruation, and injury among figure skaters warrant further investigation. The high rate of injury episodes highlights the need for figure skating federations at all levels to further address this issue among their female athletes.

Several limitations may have affected the generalizability of our findings. This study was cross-sectional in design, and therefore data may be subject to recall bias. Each figure skating club was asked to send four reminders, but the authors do not have data on whether this was done or not, other than the clubs answering MJ that they would forward the reminders. Skaters who participated in the study differed from the population invited according to competitive level (*p* < 0.001), but not according to sex or birthyear. Skaters from club-competitions-level were overrepresented among the respondents, while skaters from star-competitions-level were underrepresented. Participants were recruited as a convenience sample using the geographical region of the first author (MJ) and with whom she had contact before the study. Participants' answers to the questionnaire might have been biased by specific perceptions or figure skating experiences in this region.

Another limitation is that the online questionnaire was not validated to young figure skaters. However, the online questionnaire was based on questions previously used for self-report of injury and illness among young athletics athletes, football (soccer) players, and parasport athletes (Jacobsson et al., 2010, 2012; Dahlström et al., 2012; Fagher et al., 2020) and on questions from the HBSC study. This is an international research project, that since 1985/1986, has been conducted by the World Health Organization, every fourth year in 50 countries and regions across Europe, Asia and North America (Public Health Agency of Sweden, 2014). The use of self-report questionnaires may be limited by athletes' tendency to underreport eating pathology and associated physiological features, leading to false negatives and underestimating the findings (Johnson et al., 1999). There is a possibility that athletes with injuries and worse health were overrepresented among the respondents; being more motivated to contribute to further research on these issues among figure skaters. This might lead to overreporting and false-positive rates of injury and health-related issues.

Conversely, using a time-loss definition of injury, requiring lost or altered figure skating participation, might underestimate overuse injury rates (Bahr, 2009). However, while the primary outcome measure did not capture minor injuries, the secondary outcome measure included a broader range of injury problems. In this way, differences in injury determinants between severer and minor injuries could be identified. Further, more extensive studies are needed to identify causal risk factors. For future analyses, it would be of interest to perform a study on exclusively male figure skaters.

A randomized sample might have been more representative of figure skaters in Sweden. The number of respondents was small, and the results might have been different with a higher response rate. However, the southeastern region contains female figure skaters of all levels and figure skating clubs of different sizes and conformities. Thus, the sample was fairly representative of young female Swedish figure skaters. Also, this study contains a relatively large sample compared to previous studies on the community level [95 skaters in a recent Italian study (Campanelli et al., 2015), 33 in a recent Japanese study (Okamura et al., 2014), and 132 in a recent American study (Sugimoto et al., 2020)].

## Conclusion

One-third of young female Swedish competitive figure skaters had sustained a severe sports injury episode during the past year, and a fifth of the respondents reported an ongoing injury episode. For both the recent severe injuries and the current injuries, overuse was the most common cause, and the lower limbs were the most common injury locations. Older age and an increased number of skipped meals per week were associated with both types of injury episodes. Thus, long-term monotonous physical loads with increasing intensity and insufficient energy intake appear to predispose for injury in female figure skaters. A sports injury in young age may lead to persistent disability and compromise a future sports career. Further examination of injury determinants among competitive figure skaters is highly warranted.

## Data Availability Statement

The datasets presented in this article are not readily available because Ethical Review Board approval was obtained for public sharing and data presentation on group level only. This means that the data used in this study can only be used for the approved research and cannot be shared by the authors. Requests to access the datasets should be directed to moa.jederstrom@liu.se.

## Ethics Statement

Ethical approval was obtained from the Regional Ethics Committee in Linkoping, Sweden (DNR 2018/483-31). The study follows the Strengthening the Reporting of Observational Studies in Epidemiology (STROBE) guidelines and the WMA Declaration of Helsinki Ethical Principles for Medical Research Involving Human Subjects. Informed consent was obtained from all skaters and guardians of skaters younger than 15 years. The skaters could at any time withdraw their participation without stating a cause.

## Author Contributions

MJ, TT, and HG initiated and designed the overall study. MJ, TT, HG, SA, and JP designed the questionnaire. All authors were involved in the planning of the data analysis. MJ, TT, and AS performed the data preparation. AS performed the statistical analyses. MJ was responsible for writing the paper, in close collaboration with TT, HG, LK, CE, KF, SA, JP, and AS. All authors provided feedback on drafts and approved the final manuscript.

## Conflict of Interest

When initiating the study, SA and JP were part of the Medical Committee of the Swedish Figure Skating Association. SA is currently part of the Medical Committee of the Swedish Figure Skating Association. The remaining authors declare that the research was conducted in the absence of any commercial or financial relationships that could be construed as a potential conflict of interest.
