# Supplementary material for: Determinants of Sports Injury in Young Female Swedish Competitive Figure Skaters
Source: Front Sports Act Living. 2021 Jun 18;3:686019. doi: 10.3389/fspor.2021.686019 (PMC8253259; doi:10.3389/fspor.2021.686019)
Supplement: Supplementary file 1 [file Data_Sheet_1.docx]

Supplementary Material – Appendix I: Questionnaire

To guardian/guardians to skaters under 15 years:

**We as guardians live with the skater and have joint custody of the child. We hereby agree that our child is asked to participate in the study.**

**As a guardian, I have sole custody of the child. I hereby agree that my child is asked to participate in the study.**

**We, as guardians, are separated and have joint (alternate) custody of the child. I have been in contact with my child's other parent. We both agree that our child is asked to participate in the study.**

If you as guardian/s do not agree to your child being asked to participate, you can leave the questionnaire by closing down the website.

It is voluntary to participate in the study.

If you do not agree to your child being asked to participate, you do not need to state why, nor will it affect your child's future care or treatment.

To skaters (all ages):

** I hereby agree to participate in the study by answering the questionnaire.**

** No, I do not want to participate in the study.**

If you as a skater do not agree to participate in the study, you can leave the questionnaire by closing down the website.

It is voluntary to participate in the study.

If you do not want to participate, you do not have to state why, and it will not have any penalties (e.g. affect your future care or your training).

_________________________________________________________________________________

Welcome to the questionnaire for figure skaters!

Here you will be asked questions about, among other things, height, weight, how much you have exercised, how much you train now, how you feel and what help you can get if you need it. If you have suffered any injury or pain that affects your training, you will be asked to answer a few additional questions. It will take about 20 minutes to respond to the questionnaire.

The questionnaire consists of seven parts:

Part 1: Questions about you (6 items)

Part 2: Questions about your sports background and the current amount of training (7 items)

Part 3: Questions about your eating habits and sleep (11 items)

Part 4: Questions about your health (10 items)

Part 5: Figure skating questions (23 items)

Part 6: Questions about injuries and illnesses that affect your participation in figure skating (4 items)

Part 7: Questions about your Family and Friends (2 items)

It is important that you answer the questions by yourself. Some questions are about sensitive topics or experiences, which can also be sensitive to respond to together with a guardian. You do not have to answer all the questions but can complete the survey even if you skip specific questions. Participation is voluntary, and you may cancel participation at any time without explanation.

If you answer the survey on a mobile phone; feel free to turn the screen to a horizontal position to make it easier to read the questions.

*This question is also asked in the simplified questionnaire for skaters below 12 years of age.

# Part 1: Questions about you (6 items)

***Question 1.**

**Do you identify yourself as:**

 Girl

 Boy

 Other gender identity, namely: _____________

***Question 2.**

**When were you born?**

yyyy-mm

***Question 3.**

**In what country were your parents/guardians born?**

 Both parents/guardians were born in Sweden

 Both parents/guardians were born abroad

 One of my parents/guardians were born abroad

 Do not know

***Question 4.**

**What is your housing status?**

 Living with a parent/guardian

 Living with both parents/guardians

 Alternately sometimes with one, sometimes with the other parent/guardian

 Living by yourself

 Other, namely: _______________ (*e.g. with siblings or partner, in a family home or institution)*

***Question 5.**

**What is the highest level of education of your parents/guardians?**

Parent/guardian 1

 Primary school (and no further education)

 High school (and no further education)

 University or college education

 Do not know

Parent/guardian 2

 Primary school (and no further education)

 High school (and no further education)

 University or college education

 Do not know

**Question 6.**

**What is the financial situation in your family?**

 It is very good

 It is pretty good

 There are some financial difficulties

 There are major financial difficulties

 Do not know

# Part 2. Questions about your sports background and the current amount of training (7 items)

***Question 1.**

**About how old were you when you started figure skating?** *Enter your age with numbers, e.g. 6 years.*

***Question 2.**

**Why did you start figure skating?**

***Question 3.**

**Have either of your parents trained or competed in figure skating?**

 No.

 Yes, one of my parents

 Yes, both my parents

***Question 4.**

**Do you have any siblings or other relatives who train/compete in figure skating or have trained/competed in figure skating?** *You can select several options.*

 No.

 Yes, one or more older sisters

 Yes, one or more older brothers

 Yes, one or more younger sisters

 Yes, one or more younger brothers

 Yes, cousin (s)

 Yes, grandma

 Yes, grandpa

 Yes, aunt

 Yes, uncle

 Other, namely: __________________

***Question 5.**

**Do you train and/or compete in any sports other than figure skating?**

 No.

 Yes, namely: ___________

***Question 6.**

**In total, how often do you train so that you become short of breath and sweat, outside of school hours?**

 Every day

 4-6 days per week

 2-3 days per week

 1 day per week

 1-3 times a month

 Less than once a month

 Never

***Question 7.**

**In total, how many hours a week do you train so that you become short of breath and sweat, outside of school hours?**

 About 30 minutes

 About 1 hour

 About 2-3 hours

 About 4-6 hours

 About 7-9 hours

 About 10-12 hours

 About 13-15 hours

 About 16-18 hours

 About 19-21 hours

 About 21-23 hours

 About 24-26 hours

 26 hours or more

# Part 3: Questions about your eating habits and sleep (11 items)

***Question 1.**

**Weekdays, how many days/week do you usually eat…?**

|  | Never | 1 | 2 | 3 | 4 | 5 |
| --- | --- | --- | --- | --- | --- | --- |
| Breakfast |  |  |  |  |  |  |
| Lunch |  |  |  |  |  |  |
| Dinner |  |  |  |  |  |  |

**Weekends, how many days/week do you usually eat…?**

|  | Never | One of the days | Both days |
| --- | --- | --- | --- |
| Breakfast |  |  |  |
| Lunch |  |  |  |
| Dinner |  |  |  |

***Question 2.**

**Weekdays, how many days/week do you usually eat snacks?**

| Never | 1 | 2 | 3 | 4 | 5 | More than once a day |
| --- | --- | --- | --- | --- | --- | --- |
|  |  |  |  |  |  |  |

**Weekends, how many days/week do you usually eat snacks?**

| Never | One of the days | Both days | More than once a day |
| --- | --- | --- | --- |
|  |  |  |  |

***Question 3.**

**How often do you usually eat/drink…?**

Fruit

 Never

 Less than once a week

 Once a week

 2-4 days a week

 5-6 days a week

 Every day, once a day

 Every day, several times a day

Vegetables

 Never

 Less than once a week

 Once a week

 2-4 days a week

 5-6 days a week

 Every day, once a day

 Every day, several times a day

Sweets *(e.g. sweets, chips, chocolate, cakes)*

 Never

 Less than once a week

 Once a week

 2-4 days a week

 5-6 days a week

 Every day, once a day

 Every day, several times a day

Sweet drinks *(e.g. Coca-Cola, juice, milk)*

 Never

 Less than once a week

 Once a week

 2-4 days a week

 5-6 days a week

 Every day, once a day

 Every day, several times a day

**Question 4.**

**Do you take any supplements?** *Dietary supplements are foods to supplement the usual food; e.g. sports drinks, nutritional drinks, energy bars, protein powder.*

 Yes

 No.

 Do not know

If yes: which ones?

 Nutritional drink

 Energy drinks (e.g. Red Bull and similar drinks)

 Energy bars

 Protein powder

 Vitamin tablets

 Mineral tablets

 Other, namely _______________

Why do you take supplements? *You can select more than one option.*

 To get more energy

 To protect against disease and / or injury

 To enhance physical performance

 To compensate for an inadequate diet

 Other, namely: ______________________________________

**Question 5.**

**Are you dieting or are you doing something else to lose weight right now?**

 No, I weigh just enough

 No, but I need to lose weight

 No, but I need to gain weight

 Yes

If so, why?

***Question 6.**

**Are you worried about your weight?**

 Yes, I am careful not to gain weight

 Yes, I am careful not to lose weight

 No, I do not care if I gain or lose weight

***Question 7.**

**Have you been requested to change your weight?**

 Yes, to gain weight

 Yes, to lose weight

 No.

7a. If yes, to gain weight, who asked you to? *You can select several options.*

 Parent

 Coach

 Another family member

 Companion

 Other

Why were you asked to gain weight? *You can select several options.*

 To improve my health

 To change my appearance

 To be able to perform better in figure skating

 Other, namely: _______________________________

7b. If yes, to lose weight, who asked you to? *You can select several options.*

 Parent

 Coach

 Another family member

 Companion

 Other

Why were you asked to lose weight? *You can select several options.*

 To improve my health

 To change my appearance

 To be able to perform better in figure skating

 Other, namely: _______________________________

***Question 8.**

**What time do you usually go to bed when you go to school/work the next morning?** *State when you usually go to bed.*

 Before 20.00

 20.00

 20.30

 21.00

 21.30

 22.00

 22.30

 23.00

 23.30

 24.00

 00.30

 01.00

 01.30

 02.00

 Later

***Question 9.**

**What time do you usually get up on the days you go to school/work?** *State when you usually get up.*

 Before 05.00

 05.00

 05.30

 06.00

 06.30

 07.00

 07.30

 08.00

 Later

***Question 10.**

**What time do you usually go to bed at weekends/holidays?** *State when you usually go to bed.*

 Before 20.00

 20.00

 20.30

 21.00

 21.30

 22.00

 22.30

 23.00

 23.30

 24.00

 00.30

 01.00

 01.30

 02.00

 02.30

 03.00

 03.30

 04.00

 Later

***Question 11.**

**What time do you usually get up during weekends/holidays?** *State when you usually get up.*

 Before 05.00

 05.00

 05.30

 06.00

 06.30

 07.00

 07.30

 08.00

 08.30

 09.00

 09.30

 10.00

 10.30

 11.00

 11.30

 12.00

 12.30

 13.00

 Later

# Part 4: Questions about your health (10 questions)

***Question 1.**

**How tall are you?** *Without shoes.*

___cm

***Question 2.**

**If you are still growing, how much have you grown in cm in the last 12 months?**

___ cm

***Question 3.**

**What do you weigh?** *Without clothes.*

___kg

***Question 4.**

**If you identify as a girl, have you had your first period?**

 No

 Yes

4a. If yes, at what age?

___year

 Do not remember

4b. If yes, is your period regular?

 No

 Yes

***Question 5.**

**Do you have a long-term illness, disability or other long-term health problem that have been diagnosed by a doctor?** *For example, allergy, asthma, ADHD, ASD/Asperger, diabetes.*

 Yes

 No 🡪 go to question xx

5a. If yes, what disease or other long-term health problem do you have? *You can select several options.*

 Diabetes

 Allergy

 Eczema

 ADHD

 ASD/Asperger

 Other, namely: __________________________________

***Question 6.**

**How do you think your health is today?**

 Very good

 Good

 Pretty bad

 Poor

***Question 7.**

**Have you used any of the following drugs in the last month?** *You can select several options.*

 Painkillers (e.g. Alvedon, Panodil)

 Anti-inflammatory drugs (e.g. Ipren, Voltaren)

 Medicines for allergies

 If you are a girl, contraceptives

 Other medicines

 No, I have not used any drugs in the last month.

7a. Painkillers

 Yes, once

 Yes, more than once

7b. Anti-inflammatory drugs

 Yes, once

 Yes, more than once

7c. Medicines for allergies

 Yes, once

 Yes, more than once

7d. Contraceptives

 Yes, according to the usual prescription on the packaging

 Yes, according to special prescription

7e. Other drugs

What other medicines have you used in the last month? Write also how often you have used these (once or more than once in the last month).

***Question 8.**

**Below are a number of statements that people have used to describe how they feel. Read each statement and tick the statement that fits how you usually feel. There are no right or wrong answers. Do not think too much about any statement but answer as you think best suits how you usually feel.**

Usually, I feel calm

 Not at all

 Quite a bit

 Pretty much

 A lot

Usually, I am tense

 Not at all

 Quite a bit

 Pretty much

 A lot

Usually, I feel upset

 Not at all

 Quite a bit

 Pretty much

 A lot

Usually, I am relaxed

 Not at all

 Quite a bit

 Pretty much

A lot

Usually, I feel content

 Not at all

 Quite a bit

 Pretty much

A lot

Usually, I am worried

 Not at all

 Quite a bit

 Pretty much

A lot

***Question 9.**

**Do you think that you are ...?**

 far too skinny

 a little too skinny

 normal

 a little too fat

 far too fat

***Question 10.**

**For each statement, mark what is closest to how you have felt over the last 2 weeks.**

I have felt cheerful and in good spirits

 All the time

 Mostly

 More than half the time

 Less than half the time

 Sometimes

 Never

Skip question

I have felt calm and relaxed

 All the time

 Mostly

 More than half the time

 Less than half the time

 Sometimes

 Never

Skip question

I have felt active and vigorous

 All the time

 Mostly

 More than half the time

 Less than half the time

 Sometimes

 Never

Skip question

I woke up feeling fresh and rested

 All the time

 Mostly

 More than half the time

 Less than half the time

 Sometimes

 Never

Skip question

My daily life has been filled with things that interest me

 All the time

 Mostly

 More than half the time

 Less than half the time

 Sometimes

 Never

Skip question

# Part 5: Figure skating questions (23 questions)

***Question 1.**

**In which club do you practice figure skating?** *Choose a club.*

Apladalens Konståkningsförening

IK Graip

Jönköpings Skridskoklubb

Kalmar Konståkningsklubb

Linköpings Konståkningsförening

Norrköpings Konståkningsklubb

Nässjö Skridsko- och Konståkningsklubb

Rosenlunds Konståkningsförening

Växjö Konståkningsklubb

***Question 2.**

**In which discipline(s) do you train or compete?** *You can select several options.*

 Singles skating

 Team skating

 Pair skating

 Ice dance

***Question 3.**

**Which tests have you accomplished?** *Mark the tests you have accomplished.*

 Competition test

Basic test

 Basic test Basic level

 Basic 1

 Basic 2

 Basic 3

 Basic 4

Free-skating test

 Free-skating test Basic level

 Free-skating test 1

 Free-skating test 2

 Free-skating test 3

 Free-skating test 4

 Free-skating test 5

 Free-skating test 6

 Free-skating test 7

***Question 4.**

**Which competition class are you in right now?**

Elite series

 Novice 13 A

 Novice 15 A

 Junior A

 Senior A

 Do not know

A-competition

 Minor A

 Novice 13 A

 Novice 15A

 Junior A

 Senior A

 Do not know

Club competition

 Minor B

 Novice 13 B

 Novice 15 B1

 Novice 15 B2

 Junior B1

 Junior B2

 Senior B1

 Senior B2

 Do not know

Star competition

 Minor star class

 Novice 13 star class

 Novice 13 star class G

 Novice 15 star class

 Novice 15 star class G

 Junior star class

 Junior star class G

 Senior star class

 Senior star class G

 Do not know

 Adults

***Question 5.**

**Have you participated in your first figure skating competition yet?**

 No

 Yes

If yes, about how old were you when you competed in figure skating for the first time?

_____years old

 Do not remember

***Question 6.**

**Have you landed your first double jump?**

 No

 Yes

If yes, about how old were you when you landed your first double jump?

_____years old

 Do not remember

***Question 7.**

**Have you landed your first triple jump?**

 No

 Yes

If yes, about how old were you when you landed your first triple jump?

_____years old

 Do not remember

***Question 8.**

**Which jumps have you landed?** *You can select several options*

 1 Salchow

 1 Toeloop

 1 Loop

 1 Flip

 1 Lutz

 1 Axel

 2 Salchow

 2 Toeloop

 2 Loop

 2 Flip

 2 Lutz

 2 Axel

 3 Salchow

 3 Toeloop

 3 Loop

 3 Flip

 3 Lutz

 3 Axel

About how old were you when you landed 1A?

_____years old

 Do not remember

About how old were you when you landed 2A?

_____years old

 Do not remember

***Question 9.**

**How many different coaches have you had in total in the last year?** *Add up all the different coaches you have had in, for example, ice, fitness training, dance, choreography, mental training. Enter the number of coaches in numbers.*

___ different coaches

***Question 10.**

**Have you changed clubs in the last year?**

 No

 Yes

10a. If yes, how many times have you changed clubs? *Enter the number in numbers.*

10b. If yes, why did you change clubs?

***Question 11.**

**In which direction do you jump and spin?**

 Right (= lands on left foot)

 Left (= lands on the right foot)

 Both

 Do not know

11a. Are you right-handed or left-handed?

 Right

 Left

 Both

 Do not know

11b. With which foot do you kick a ball?

 Right

 Left

 Both

 Do not know

***Question 12.**

**On average, how many hours a week have you practised figure skating in the last 12 months?** *Enter the number of hours in numbers.*

___ hours/week

***Question 13.**

**On average, how many sessions a week have you practised figure skating in the last 12 months?** *Enter the number of hours in numbers.*

___ pass/week

***Question 14.**

**How many competitions have you participated in in the last 12 months?** *Enter the number of competitions in numbers.*

___ number of competitions

***Question 15.**

**On average, how many days per week have you rested completely from sports in the last 12 months?** *Enter the number of resting days in numbers*.

___ days / week

***Question 16.**

**On average, how long does a workout usually last?** *Including warming up and stretching/cooling down. Choose the option that suits you best.*

Session on ice

 30 minutes

 1 hour

 1.5 hours

 2 hours

 2.5 hours

 3 hours

Fitness training session

 30 minutes

 1 hour

 1.5 hours

 2 hours

 2.5 hours

 3 hours

 Do not have fitness training

Dance session

 30 minutes

 1 hour

 1.5 hours

 2 hours

 2.5 hours

 3 hours

 Do not have dancing

Other, state which: _______________________ (e.g. yoga or choreography)

 30 minutes

 1 hour

 1.5 hours

 2 hours

 2.5 hours

 3 hours

***Question 17.**

**Do you usually warm up before a workout on ice?**

 No.

 Yes

If so, how long do you usually warm-up?

 Less than 15 minutes

 15 minutes

 30 minutes

 45 minutes

 1 hour

 1.5 hours

 2 hours

***Question 18.**

**Do you usually cool down and stretch after a workout on ice?**

 No.

 Yes

If so, how long do you usually jog down and stretch?

 Less than 15 minutes

 15 minutes

 30 minutes

 45 minutes

 1 hour

 1.5 hours

 2 hours

***Question 19.**

**Do you use any form of protection/shock absorption during training?** *You can select several options.*

 No.

 Yes, headband

 Yes, helmet

 Yes, knee pads

 Yes, jumping pants

 Yes, namely: ________________________

***Question 20.**

**If you do not use protection during training, why not?** *You can select several options.*

 It is not needed

 It limits me on the ice

 It is not convenient

 It's not good looking

 Other, namely: __________________________

***Question 21.**

**Do you use any type of aids when training?** *For example, cooling spray, bungapads, inserts in the skates.*

 No.

 Yes

If so, what type of aids do you use?

*** Question 21b.**

**If you use inserts, why do you have them?** *You can select several options.*

 To provide support for the arch of the foot

 To increase comfort

 Due to too large skate

 Other, namely: _______________________

***Question 22.**

**What brand are your skates?**

 Edea

 Risport

 Wifa

 Jackson

 Other, namely: ______________

***Question 23.**

**Have you customized your skates in any way?** *For example, custom-made shoe, pushed out at the toe or made room for ankle(s)/malleolus.*

 No.

 Yes

If so, in what way have you adapted your skates?

# Part 6: Questions about injuries and illnesses that affect your participation in figure skating (4 questions)

***Question 1.**

**In the last 12 months, have you suffered an injury/pain in connection with training or competition that prevented you from fully participating in training or competition for at least three weeks?**

 Yes

 No 🡪 go to question xx

Skip question

1a. How did the injury/pain arise? If you have had several severe injuries, describe the most important one.

 The injury/pain occurred as a result of external force on one occasion (e.g. got stuck with the foot in the ice during jumping).

 The injury/pain arose through repeated overload without a single clear cause.

 The injury/pain arose through repeated overload with a known cause.

Skip question

1b. Describe where in the body the injury/pain was located *(select one or more options)*;

 Hip

 Groin

 Thigh

 Knee

 Lower legs (front or sides)

 Calf

 Achilles tendon

 Ankle

 Foot

 Toes

 Head

 Neck

 Thoracic spine

 Lumbar spine

 Shoulder

 Other

Skip question

1c. On which side of the body was the injury/pain in [xxx] localized? *State right left or both.*

Skip question

1d. Did you seek medical help for the injury/pain in [xxx]?

 Yes

 No

Skip question

1e. What medical help did you seek for the injury/pain in [xxx]?

Skip question

***Question 2.**

**Are you currently bothered by an injury/pain that has arisen in connection with training or competition in figure skating that prevents you from fully participating in training or competition?**

 Yes

 No 🡪 go to question xx

Skip question

2a. How did the injury/pain arise? If you have multiple injuries, describe the most important one.

 The injury/pain occurred as a result of external force on one occasion (e.g. got stuck with the foot in the ice during jumping).

 The injury/pain arose through repeated overload without a single clear cause.

 The injury/pain arose through repeated overload with a known cause.

Skip question

2b. Describe where in the body the injury/pain is located (select one or more options);

 Hip

 Groin

 Thigh

 Knee

 Lower legs (front or sides)

 Calf

 Achilles tendon

 Ankle

 Foot

 Toes

 Head

 Neck

 Thoracic spine

 Lumbar spine

 Shoulder

 Other

Skip question

2c. On which side of the body is the injury/pain in [xxx] localized? *State right left or both.*

Skip question

2d. Have you sought medical help for the injury/pain in [xxx]?

 Yes

 No

Skip question

2e. What medical help have you sought for the injury/pain in [xxx]?

Skip question

***Question 3.**

**In the last 12 months, due to illness, have you not been able to fully participate in training or competition for at least three weeks?**

 Yes

 No 🡪 go to question xx

Skip question

3a. If you have been prevented by an illness/health problem, state which;

 Infection of the respiratory tract

 Gastrointestinal infection

 Chronic illness/health problems (stated in part 4 of the questionnaire)

 Other, namely ________________________

Skip question

3b. Did you seek medical help for this disease/health problem?

 Yes

 No

Skip question

3c. If yes, what medical help did you seek for the disease/health problem?

Skip question

***Question 4.**

**Are you currently suffering from an illness that prevents you from fully participating in training or competition in figure skating?**

 Yes

 No 🡪 go to question xx

Skip question

4a. If you have been prevented by an illness/health problem, state which;

 Infection of the respiratory tract

 Gastrointestinal infection

 Chronic illness/health problems (stated in part 4 of the questionnaire)

 Other, namely ________________________

Skip question

4b. Have you sought medical help for the disease/health problem?

 Yes

 No

Skip question

4c. What medical help have you sought for the disease/health problem?

Skip question

# Part 7: Questions about Your Family and Friends (2 Questions)

**Question 1.**

**We are interested in how you experience the following statements.** *Read each statement carefully. Mark how well you agree with each statement.*

My family is really trying to help me

Do not agree at all – 1 – 2 – 3 – 4 – 5 – 6 – 7 – Agree fully

My family gives me the emotional support and help I need

Do not agree at all – 1 – 2 – 3 – 4 – 5 – 6 – 7 – Agree fully

I can talk about my problems with my family

Do not agree at all – 1 – 2 – 3 – 4 – 5 – 6 – 7 – Agree fully

My family helps me make decisions

Do not agree at all – 1 – 2 – 3 – 4 – 5 – 6 – 7 – Agree fully

My friends are really trying to help me

Do not agree at all – 1 – 2 – 3 – 4 – 5 – 6 – 7 – Agree fully

I can count on my friends when something goes wrong

Do not agree at all – 1 – 2 – 3 – 4 – 5 – 6 – 7 – Agree fully

I can share joy and sorrow with my friends

Do not agree at all – 1 – 2 – 3 – 4 – 5 – 6 – 7 – Agree fully

I can talk about problems with my friends

Do not agree at all – 1 – 2 – 3 – 4 – 5 – 6 – 7 – Agree fully

***Question 2.**

**Below are questions about bullying.** *A skater is bullied when another skater or a group of skater says or does nasty or unpleasant things towards him/her. It is also bullying when a skater is repeatedly teased in a way that he/she does not like or if he/she is left out.*

Have you been bullied in connection with figure skating?

 Yes

 No

Have you been involved in bullying others in connection with figure skating?

 Yes

 No
