# Supplementary material for: Determinants of Sports Injury in Young Female Swedish Competitive Figure Skaters
Source: Front Sports Act Living. 2021 Jun 18;3:686019. doi: 10.3389/fspor.2021.686019 (PMC8253259; doi:10.3389/fspor.2021.686019)
Supplement: Supplementary file 2 [file Data_Sheet_2.docx]

Supplementary Material – Appendix II: Box: Swedish Figure Skating 101

**Table A.** The different jumps in figure skating and their Base Value according to the International Skating Union (ISU) Grade of Execution (GOE) during the 2018-2019 season

| Jump (abbreviation) | Base Value for a double jump |
| --- | --- |
| Salchow (S) | 1.30 |
| Toeloop (T | 1.30 |
| Loop (Lo) | 1.70 |
| Flip (F) | 1.80 |
| Lutz (Lz) | 2.10 |
| Axel (A) | 3.30 |

**Table B.** Age-groups when competing in Swedish figure skating during the 2018-2019 season.

| Competitive group | Age |
| --- | --- |
| Minor | Skater should not have reached 11 years of age before the 1^st^ of July. |
| Novice 13 | Skater has to be at least 10 years old but not 13 years old before the 1^st^ of July. |
| Novice 15 | Skater has to be at least 12 years old but not 15 years old before the 1^st^ of July. |
| Junior | Skater has to be at least 13 years old but not 19 years old before the 1^st^ of July. |
| Senior | Skater has to be at least 15 years old before the 1^st^ of July. |
| Adult | Skater has to be at least 21 years of age before the 1^st^ of July. |

**Table C.** Required jumps for competitive classes in Swedish figure skating during the 2018-2019 season. To be eligible for competition, the skater must pass the Competition test. To compete in more advanced classes, the skater has to pass further tests requiring steps, jumps, spins and spirals of increasing level.

| Swedish competitive class | Required landed jumps |
| --- | --- |
| A-level and Elite level |  |
| Senior, Junior, Novice 15 | 2S, 2T, 2Lo, 2F, 2Lz and a jump combination of two double jumps. |
| Novice 13 and Minor | Two out of 2S, 2T, 2Lo, 2F, 2Lz |
| Club competitions |  |
| Senior B1, Junior B1, Novice 15 B1 | Two out of 2S, 2T, 2Lo, 2F, 2Lz |
| Senior B2, Junior B2, Novice 15 B2, Novice 13 B, Minor B | 1F, 1Lz, 1A |
| Star Competitions |  |
| Senior 1, Junior 1, Novice 15 1, Novice 13 1 | 1S, 1T, 1Lo |
| Senior G, Junior G, Novice 15 G, Novice 13 G, Minor | No required jumps. |
| Adult | No required jumps. |
