# Supplementary material for: Determinants of Sports Injury in Young Female Swedish Competitive Figure Skaters
Source: Front Sports Act Living. 2021 Jun 18;3:686019. doi: 10.3389/fspor.2021.686019 (PMC8253259; doi:10.3389/fspor.2021.686019)
Supplement: Supplementary file 3 [file Data_Sheet_3.docx]

Supplementary Material – Appendix III: Study respondents (n=142) and non-respondents (n=258) displayed by birthyear, sex, and competitive level

**Appendix II.** Study respondents (n=142) and non-respondents (n=258) displayed by birthyear, sex, and competitive level. p-values represent the significance of comparisons between respondents and the population of figure skaters in southeastern Sweden (N = 400).

|  | Population, n (%) | Respondents, n (%) | Non-respondents, n (%) | p-value |
| --- | --- | --- | --- | --- |
| Sex |  |  |  | 0.203 |
| Boys | 9 (2%) | 5 (4%) | 4 (2%) |  |
| Girls | 391 (98%) | 137 (96%) | 254 (98%) |  |
| Birthyear |  |  |  | 0.173 |
| Before 2003 | 42 (11%) | 19 (13%) | 24 (9%) |  |
| 2003-2004 | 51 (13%) | 21 (15%) | 31 (12%) |  |
| 2005-2006 | 104 (26%) | 33 (23%) | 73 (28%) |  |
| 2007-2008 | 109 (27%) | 43 (30%) | 69 (26%) |  |
| 2009 or later | 94 (24%) | 26 (18%) | 69 (26%) |  |
| Competitive level* |  |  |  | <0.001 |
| Elite skater + A-competitions | 57 (14%) | 26 (18%) | 34 (13%) |  |
| Club competitions | 123 (31%) | 61 (43%) | 60 (23%) |  |
| Star competitions | 220 (55%) | 55 (39%) | 169 (64%) |  |

*****To be eligible for competition in Sweden, the skater must pass a Competition test. To compete in more advanced classes, the skater has to pass further tests requiring steps, jumps, spins and spirals of increasing level. Elite-/A-level skaters in the age groups senior, junior or novice 15 must be able to perform all double jumps and a jump combination of two double jumps, while elite-/A-level skaters in the age groups novice 13 and minor are required to land two different double jumps. Club-competition-level skaters must land all single jumps. There are no required jumps for skaters on star-competitions-level.
