# Supplementary material for: Determinants of Sports Injury in Young Female Swedish Competitive Figure Skaters
Source: Front Sports Act Living. 2021 Jun 18;3:686019. doi: 10.3389/fspor.2021.686019 (PMC8253259; doi:10.3389/fspor.2021.686019)
Supplement: Supplementary file 4 [file Data_Sheet_4.docx]

Supplementary Material – Appendix IV: Figure skating specific descriptive data on skate brand, length of ice-skating sessions, warm-up, cooling down/stretching, the use of protection/shock absorption, use of aids, customized skates, age when starting with figure skating, participation in other sports and competitions.

**Appendix III.** Figure skating specific descriptive data on skate brand, length of ice-skating sessions, warm-

up, cooling down/stretching, the use of protection/shock absorption, use of aids, customized skates, age

when starting with figure skating, participation in other sports and competitions (n = 137).

|  | n (%) |
| --- | --- |
| Skate brand* |  |
| Edea | 112 (82%) |
| Jackson | 14 (10%) |
| Other | 10 (7%) |
| Length of ice-skating session |  |
| 1 hour | 82 (60%) |
| 1.5 hours | 41 (30%) |
| ≥2 hours | 14 (10%) |
| Warming up before an ice-skating session |  |
| No | 30 (22%) |
| Yes | 107 (78%) |
| *Time spent warming up* *(n = 107)* |  |
| <15 minutes | 40 (37%) |
| ≥15 minutes | 67 (63%) |
| Cooling down/stretching after an ice-skating session |  |
| No | 83 (61%) |
| Yes | 54 (39%) |
| *Time spent cooling down/stretching* *(n = 54)* |  |
| <15 minutes | 24 (44%) |
| ≥15 minutes | 30 (56%) |
| Use of protection/shock absorption** |  |
| Yes | 46 (34%) |
| No | 90 (66%) |
| *Type of protection/shock absorption used (multiple options possible, n = 49)* |  |
| Headband or helmet | 32 (65%) |
| Kneepad | 7 (14%) |
| Other | 10 (21%) |
| Use of aids | |
| Yes | 53 (39%) |
| No | 84 (61%) |
| *Type of aid being used (multiple options possible, n = 69)* | |
| Bungapads | 40 (58%) |
| Gel plates or foam rubber for malleolus/bumps on the feet | 7 (10%) |
| Special soles | 14 (20%) |
| Other | 8 (12%) |
| Customized skates |  |
| No | 123 (90%) |
| Yes | 14 (10%) |
| Age when starting with figure skating |  |
| ≤4 years old | 32 (23%) |
| 5-6 years old | 55 (40%) |
| 7-8 years old | 30 (22%) |
| 9-10 years old | 15 (11%) |
| ≥11 years old | 5 (4%) |
| Practicing other sports |  |
| No | 89 (65%) |
| Yes | 48 (35%) |
| Number of competitions the skater has participated in, in the last 12 months*** |  |
| No competitions this year | 5 (4%) |
| 1-2 | 14 (10%) |
| 3-5 | 68 (50%) |
| ≥6 | 49 (36%) |

*One missing response

**One missing response

***One missing response
